# Cost of Sickness

**Published:** 1919-06

**Authors:** 


### Cost of Sickness.

The State Health Department of Texas finds that an average of 10.17 days per capita per annum were lost by the people of Texas through illness during the last three years. This represents a financial loss of $10.74 per capita or 3.3 per cent of the income of the entire popluation of the State.

The expenses of caring for the sick, which are growing more burdensome all the time, are not included in the statistics of loss through sickness. When this is added the per capita cost of sickness is increased perhaps three fold. Thus the people of Texas lose about 10 per cent of their annual earnings through illness.

The immense loss of revenue, to say nothing of the suffering caused by illness, can best be impressed on the mind by a statement of this kind; in a city of 20,000 persons the annual loss is 200,000 days or 550 years time and the cost including earning capacity and expense of caring for the sick is something like $750,000 a year. This would go a long way toward building and maintaining the necessary public improvements in a city after deducting all the expense necessary to stop the ravages of preventable diseases.

There were 24.3 deaths per thousand in the State, 45 or nearly half of which were preventable diseases. This unnecessary waste of earnings and of life is nothing less than criminal on the part of the authorities. How long will it be before our people awake to the fact that "an ounce of prevention is worth a pound of cure?"

